# Lung Transplantation for Lymphangioleiomyomatosis in Japan

**DOI:** 10.1371/journal.pone.0146749

**Published:** 2016-01-15

**Authors:** Katsutoshi Ando, Yoshinori Okada, Miki Akiba, Takashi Kondo, Tomohiro Kawamura, Meinoshin Okumura, Fengshi Chen, Hiroshi Date, Takeshi Shiraishi, Akinori Iwasaki, Naoya Yamasaki, Takeshi Nagayasu, Masayuki Chida, Yoshikazu Inoue, Toyohiro Hirai, Kuniaki Seyama, Michiaki Mishima, Respiratory Failure Research Group of the Japanese Ministry of Health, Labour, and Welfare

**Affiliations:** 1 Division of Respiratory Medicine, Juntendo University, Faculty of Medicine and Graduate School of Medicine; 2-1-1 Hongo; Bunkyo-Ku; Tokyo, Japan; 2 Department of Thoracic Surgery, Institute of Development, Aging and Cancer, Tohoku University; Seiryo-machi 4–1, Aoba-ku Sendai, Miyagi, Japan; 3 Department of General Thoracic Surgery, Osaka University Graduate School of Medicine; 2–2 Yamadaoka, Suita, Osaka, Japan; 4 Department of Thoracic Surgery, Kyoto University Graduate School of Medicine, 54 Shogoin-kawahara-cho, Sakyo-ku, Kyoto, Japan; 5 Department of General Thoracic, Breast, and Pediatric Surgery, Fukuoka University School of Medicine; 7-45-1 Nanakuma, Jonan-ku, Fukuoka City, Fukuoka, Japan; 6 Division of Surgical Oncology, Department of Translational Medical Sciences, Nagasaki University Graduate School of Biomedical Sciences; 1-7-1 Sakamoto, Nagasaki, Japan; 7 Department of General Thoracic Surgery, Dokkyo Medical University; 880 Kitakobayashi, Mibu-machi, Shimotsuga-gun, Tochigi, Japan; 8 Clinical Research Center, National Hospital Organization Kinki-Chuo Chest Medical Center; 1180, Nagasonecho, Kita-Ku, Sakai, Osaka, Japan; 9 Department of Respiratory Medicine, Graduate School of Medicine, Kyoto University; Kawahara 54, Shogoin, Sakyo-ku, Kyoto, Japan; 10 Respiratory Failure Research Group from the Ministry of Health, Labour and Welfare, Japan, (Office) Department of Respiratory Medicine, Graduate School of Medicine, Kyoto University; Kawahara 54, Shogoin, Sakyo-ku, Kyoto, Japan; University of California Los Angeles, UNITED STATES

## Abstract

**Background:**

Lung transplantation has been established as the definitive treatment option for patients with advanced lymphangioleiomyomatosis (LAM). However, the prognosis after registration and the circumstances of lung transplantation with sirolimus therapy have never been reported.

**Methods:**

In this national survey, we analyzed data from 98 LAM patients registered for lung transplantation in the Japan Organ Transplantation Network.

**Results:**

Transplantation was performed in 57 patients as of March 2014. Survival rate was 86.7% at 1 year, 82.5% at 3 years, 73.7% at 5 years, and 73.7% at 10 years. Of the 98 patients, 21 had an inactive status and received sirolimus more frequently than those with an active history (67% vs. 5%, p<0.001). Nine of twelve patients who remained inactive as of March 2014 initiated sirolimus before or while on a waiting list, and remained on sirolimus thereafter. Although the statistical analysis showed no statistically significant difference, the survival rate after registration tended to be better for lung transplant recipients than for those who awaited transplantation (p = 0.053).

**Conclusions:**

Lung transplantation is a satisfactory therapeutic option for advanced LAM, but the circumstances for pre-transplantation LAM patients are likely to alter with the use of sirolimus.

## Introduction

Lymphangioleiomyomatosis (LAM) is a rare neoplastic disease, characterized by the proliferation of abnormal smooth muscle-like cells (LAM cells), which lead to cystic destruction of the lungs, chylous effusions and the formation of lymphangioleiomyomas [1]. This disease is found primarily in women of childbearing age and can occur either as a sporadic disease (sporadic LAM) or as a pulmonary manifestation of tuberous sclerosis complex (TSC) (TSC-associated LAM) [2–4]. Clinical manifestations include exertional dyspnea, pneumothorax, hemoptysis, and chylous leakage into the pleural and/or peritoneal cavities. The disease continuously progresses and eventually results in respiratory failure, although the pace of progression varies considerably among LAM patients [5]. Until recently, therapeutic medical interventions for LAM were very limited; that is, only lung transplantation has been established as the definitive treatment option for patients with an advanced stage of LAM [6]. Owing to its rarity, LAM accounted for only about 1.0% of all causes for whole heart or lung transplantations in an international survey [7]. However, in Japan, advanced LAM is one of the main indicators (20%) requiring such grafts. Other candidates are sufferers with primary pulmonary hypertension (18%) or idiopathic interstitial pneumonia (18%); these patients are the most common recipients of single lung transplants from brain-dead-donors [8].

Recently, a molecular-targeting therapy for LAM has been established. LAM cells appear to result from the dysregulated mechanistic target of rapamycin (mTOR) complex 1 (mTORC1) signaling, which is a key regulatory pathway of protein synthesis, cell growth, and energy metabolism due to the *TSC* gene mutation [2–4]. Sirolimus, an mTOR inhibitor, blocks mTORC1-mediated activation and restores homeostasis [9]. A recent clinical trial, the Multicenter International Lymphangioleiomyomatosis Efficacy and Safety of Sirolimus (MILES) trial, successfully demonstrated that sirolimus stabilized lung function and improved the quality of life in patients with LAM [10]. Furthermore, our clinical review of Japanese LAM patients indicated that low-dose sirolimus yielded the clinical benefits of improving pulmonary function and decreasing chylous effusion [11]. Although sirolimus had been administered as off-label in Japan if LAM patients wish to take, its use has been approved for LAM therapy and covered by medical insurance in Japan as of December 2014. That is, sirolimus is predicted to become a common treatment strategy that will provide a huge impact on lung transplantation for LAM patients not only in Japan but also worldwide. In fact, the benefit seems to be greatest in Japan, since transplantation for LAM patients is the most frequent among various indications of underlying pulmonary diseases in Japan [8]. Therefore, the national survey conducted here to elucidate the clinical features of LAM in patients with an advanced stage of the disease and to document their outcomes after registration for lung transplantation provides a much-needed basis for foreseeing the future of this procedure in Japan.

## Materials and Methods

### Study design

From 1999 to 2013, 98 LAM patients from six lung transplant centers were registered for lung transplantation in the Japan Organ Transplantation Network. To determine their clinical characteristics at registration and their post-transplant status as of March 2014, we retrospectively reviewed their medical records and summary worksheets at registration. The data compiled included presenting features, diagnostic methods, clinical findings, pre-transplant history of sirolimus treatment, results of arterial blood gas analysis, echocardiography, right heart catheterization (RHC), six-minute walk test, and lung function tests. Post-transplant outcomes including LAM-related complications in one year after transplantation, survival times, and causes of death were analyzed. We noted the causes of deaths after lung transplantation as well as while on the waiting list.

RHC was performed at registration in 25 (25.5%) of 98 patients. The diagnosis of pre-capillary pulmonary hypertension (PH) was established based on data of RHC; which was defined as mean pulmonary artery pressure (MPAP) ≥ 25 mmHg and pulmonary artery wedge pressure (PAWP) ≤ 15 mmHg [12]. Our retrospective review, verbal consent procedure and analysis of their data in study, was approved by the ethics committee of Juntendo, Tohoku, Osaka, Kyoto, Fukuoka and Nagasaki Universities, and all patient data were anonymous. None of the transplant donors were from a vulnerable population and all donors or next of kin provided written informed consent that was freely given.

### Inactive system

The Japanese organ-transplant registration system allows recipients to self-determine whether they wish to have an “inactive status”, i.e., temporary removal from the waiting list for lung transplantation; when they prefer restoration to an “active status”, they can resume their position on the waiting list where they were originally registered. Common reasons for an inactive status to postpone transplantation include: 1) the prospective recipient has been stable with medical care although severely disabled, and 2) the recipient is anxious and fears the operation. To determine the characteristics of patients who choose an inactive status, we explored the specific reasons for being an inactive status from medical records, classified all 98 patients as with or without that history, and then compared their clinical data.

### Lung function tests

Lung function tests were performed in each institution according to American Thoracic Society standards. Forced vital capacity (FVC), forced expiratory volume in 1 second (FEV_1_), diffusing capacity for carbon monoxide (DL_CO_) and DL_CO_ per unit of alveolar volume (DL_CO_/VA) for each patient were expressed as a percentage of the predicted values (FVC %predicted, FEV_1_%predicted, DL_CO_ %predicted and DL_CO_/VA %predicted). The reference values were obtained from the prediction equations of the Japanese Respiratory Society [13].

### Statistical analysis

We used the *Chi*-squared or Mann-Whitney tests, where appropriate, to compare patients’ characteristics. Correlations of MPAP with clinical parameters were evaluated by Spearman’s rank correlation analysis. Survival rates were estimated using the Kaplan-Meier method. For all statistical analyses, we used the SPSS, version 21, and a p value less than 0.05 was considered significant.

## Results

### Patients’ characteristics at the registration for lung transplantation

All patients were female, and their mean ages at the onset of symptoms and at the diagnosis were 32.1 and 34.2 years, respectively. Ninety patients (92%) had been diagnosed with sporadic LAM, whereas eight had TSC-associated LAM. The diagnosis of LAM was established by histopathological examination (n = 81), by cytological examination of chylous fluid (n = 1), or by the combined findings of characteristic computed tomography (CT) and/or elevated serum VEGF-D (> 800 pg/mL) (n = 8) (see S1 Table for detailed clinical characteristics at the diagnosis of LAM).

The mean age at registration for lung transplantation was 39.5 years (Table 1); the mean period from diagnosis to registration was 5.3 years. At registration, 21 patients (21%) had chylous effusion; nine had both chylothorax and ascites, whereas eight had chylothorax alone and four had chylous ascites alone. Twenty-two patients (22%) had renal angiomyolipomas. Lymphangioleiomyomas along axial lymphatics were suspected from imaging studies of fifty patients (51%): 14 (14%) had abdominal lymphadenopathies, two (2%) had pelvic lymphadenopathies, and 34 (35%) had both abdominal and pelvic lymphadenopathies.

**Table 1:** Clinical characteristics of 98 LAM patients at registration for lung transplantation

Variable	Value
Age at registration for lung transplantation -yr (range)	39.5 ± 7.3 (24–59)
Clinical features at registration—N. (%)	
Patients with lung involvement alone	28 (29)
Patients with extrapulmonary manifestation	70 (71)
Chylous effusion	21 (21)
Chylothorax	17 (17)
Ascites	13 (13)
Mediastinal lymphadenopathy	7 (7)
Angiomyolipoma	22 (22)
Retroperitoneal lymphagioleiomyoma	48 (49)
Pelvic lymphangioleiomyoma	36 (37)
Lymph edema	6 (6)
Body mass index–(range)	19.2 ± 3.6 (13.4–29.3)
Modified MRC classification -N (%)	
I	1 (1)
II	41 (42)
III	47 (48)
IV	8 (8)
Unknown	1 (1)
Pulmonary function (range)	
FVC (L)	2.11 ± 0.72 (0.57–3.88)
FVC (%predicted)	70.3 ± 24.3 (18.5–124.7)
FEV_1_ / FVC	40.2 ± 15.2 (15.6–91.5)
FEV_1_ (L)	0.85 ± 0.45 (0.22–2.14)
FEV_1_ (%predicted)	32.8 ± 17.0 (9.1–77.5)
DLco (%predicted)	24.7 ± 11.2 (0.7–58.3)
DLco/VA (%predicted)	24.1 ± 13.6 (0.3–73.6)
Six minutes walking test (range)	
Distance (m)	252.0 ± 89.4 (58–440)
Lowest SpO_2_ (%)	84.7 ± 5.9 (67–96)
Arterial blood gas (room air, N = 71) *	
pH (range)	7.42 ± 0.02 (7.37–7.47)
PaO_2_ (Torr) (range)	55.7 ± 8.7 (31.6–73.4)
PaCO_2_ (Torr) (range)	39.0 ± 5.3 (29.4–54.3)

Plus-minus data are presented as means ± SD.

* Of 98 LAM patients, 71 had the data of arterial blood gas analysis at room air.

Abbreviations used are: DL_CO_, carbon monoxide diffusing capacity; DL_CO_/VA, DL_CO_ per alveolar gas volume; FEV_1_, forced expiratory volume in one second; FVC, forced vital capacity; and %predicted, a percentage of the predicted values.

All patients were severely disabled at the time of registration. More than half (56/98 = 57%) showed dyspnea of modified MRC grade III or more. As Table 1 depicts, mean values of FVC, FEV_1_, DL_CO_, PaO_2_, and six-minute-walking distance (6MWD) at registration were 2.11 L (70.3%predicted), 0.85 L (32.8%predicted), 24.7%predicted, 55.7 Torr, and 252 m, respectively. Ninety-four patients (96%) utilized supplemental oxygen at registration.

### Haemodynamics at registration for lung transplantation

Data from echocardiography were available for all patients, and 16 (16%) showed an estimated systolic pulmonary arterial pressure (SPAP) ≥ 40 mmHg (Table 2). The results of RHC in 25 patients yielded mean values for SPAP, MPAP and pulmonary vascular resistance (PVR) of 31.5 mmHg, 22.8 mmHg and 256.0 dyne sec/cm^5^, respectively. Nine of 25 patients (36%) had MPAP ≥ 25 mmHg, and pre-capillary PH was diagnosed in eight patients (one patient had PAWP ≥ 15 mmHg). The correlation between MPAP and clinical parameters was examined in 25 LAM patients who underwent RHC (S2 Table). MPAP had a statistically significant negative correlation with DL_CO_ %predicted (r = -0.654, p = 0.001), but not with other clinical parameters including PaO_2_ (r = -0.348, p = 0.223) and FEV_1_%predicted (r = 0.283, p = 0.181). Among 25 LAM patients who had RHC at registration, the actual values of SPAP estimated by echocardiography were available in only 13 patients; results for the remaining 12 patients were just descriptions, e.g., “no pulmonary hypertension.” When the estimated SPAP at echocardiography and MPAP at RHC in the 13 patients were analyzed, we found a statistically significant correlation between them (S1 Fig; r = 0.589, p = 0.034). All six patients with estimated SPAP ≥ 40 mmHg at echocardiography also had MPAP ≥ 25 mmHg at RHC.

**Table 2:** Haemodynamic data at registration for lung transplantation

Variable	Value
Echocardiography-n (%)	
Estimated SPAP ≥ 40 mmHg	16 (16)
Estimated SPAP ≥ 50 mmHg	3 (3)
Right heart catheterization (n = 25)	
Heart rate (beats per min)	78.9 ± 13.9 (61–109)
Mean right atrial pressure (mmHg)	4.9 ± 2.8 (1–12)
SPAP (mmHg)	31.5 ± 9.4 (20–50)
MPAP (mmHg)	22.8 ± 6.8 (13–35)
Patients with ≥ 20 mmHg-n (%)	12 (48)
Patients with ≥ 25 mmHg-n (%)*	9 (36)
Mean PCWP (mmHg)	8.1 ± 4.0 (2–20)
Pulmonary vascular resistance (dyne sec/cm^5^)	256.0 ± 89.0 (119.0–442.9)
Cardiac index (L/min/m^2^)	3.2 ± 0.7 (2.0–5.1)

Plus-minus data are presented as means ± SD.

* Of 9 LAM patients, eight patients had mean PCWP < 15 mmHg.

Abbreviations used are: MPAP, mean pulmonary artery pressure; SPAP, systolic pulmonary artery pressure; and PCWP, pulmonary capillary wedge pressure.

### Lung transplantation

The status of 98 LAM patients (as of March 2014) who were registered for lung transplantation is summarized in Table 3. Transplantation was performed in 57 patients (58%); 53 patients received lungs from brain-dead-donors (single lung transplantation in 48 patients and double in 5 patients), whereas four received lobar lungs from living-donors. Mean ages at registration and at transplantation were 39.0 and 41.8 years, respectively. Eight of 57 patients (14%) who were transplanted had a history of inactive status while they waited: i.e., an inactive period (median) of 170 days (range; 48–1,624 days). Duration from registration to transplantation (median) was 969 days (range; 29–3,381 days).

**Table 3:** The status of 98 LAM patients who were registered for lung transplantation (as of March 2014).

Status	
Patients who had been transplanted -n (%)	57 (58)
Age at registration -yr (range)	39.0 ± 7.8 (24–59)
Age at lung transplantation -yr (range)	41.8 ± 8.2 (24–61)
Days from registration to transplantation -median (range)	969 (29–3,381)
History of inactive status on waiting list -n (%)	8 (14)
days of inactive on waiting list -median (range)	170 (48–1,624)
Transplantation	
Deceased-donor lung transplantation—n (%)	53 (93)
Single / Bilateral	48 (91) / 5 (9)
Living-donor lung transplantation—n (%)	4 (7)
Patients on waiting list -n	32 (33)
Active on waiting list—n (%)	20 (20)
History of inactive on waiting list—n (%)	0
Cumulative days from registration -median (range) *	414 (96–1,179)
Inactive on waiting list—n (%)	12 (12)
Cumulative days from registration -median (range)*	2,153 (942–3,461)
Patients who died during waiting term	9 (9)
Days from registration -median (range)	313 (132–3,121)
History of inactive status on waiting list—n (%)	1 (1) **
Cause of death	
Respiratory failure	8 (8)
Hemoptysis	1 (1)

* Median days from registration to March 2014

** The patient who died 3,121 days after registration had a history of 1,598 days of inactive status during on the waiting list. If this patient was excluded from this analysis, the median days from registration was 308 and the range was 132–1,816 days.

Thirty-two patients (33%) were still on the waiting list; twenty patients (20%) were in active status (the median waiting period after registration, 414 days), whereas twelve (12%) remained in the inactive group (the median waiting period after registration, 2,153 days). Nine patients (9%) died while on the waiting list (313 days after registration [median]), and one of them had a history of inactive status (Table 3).

### Outcomes of lung transplantation

**Fig 1:**
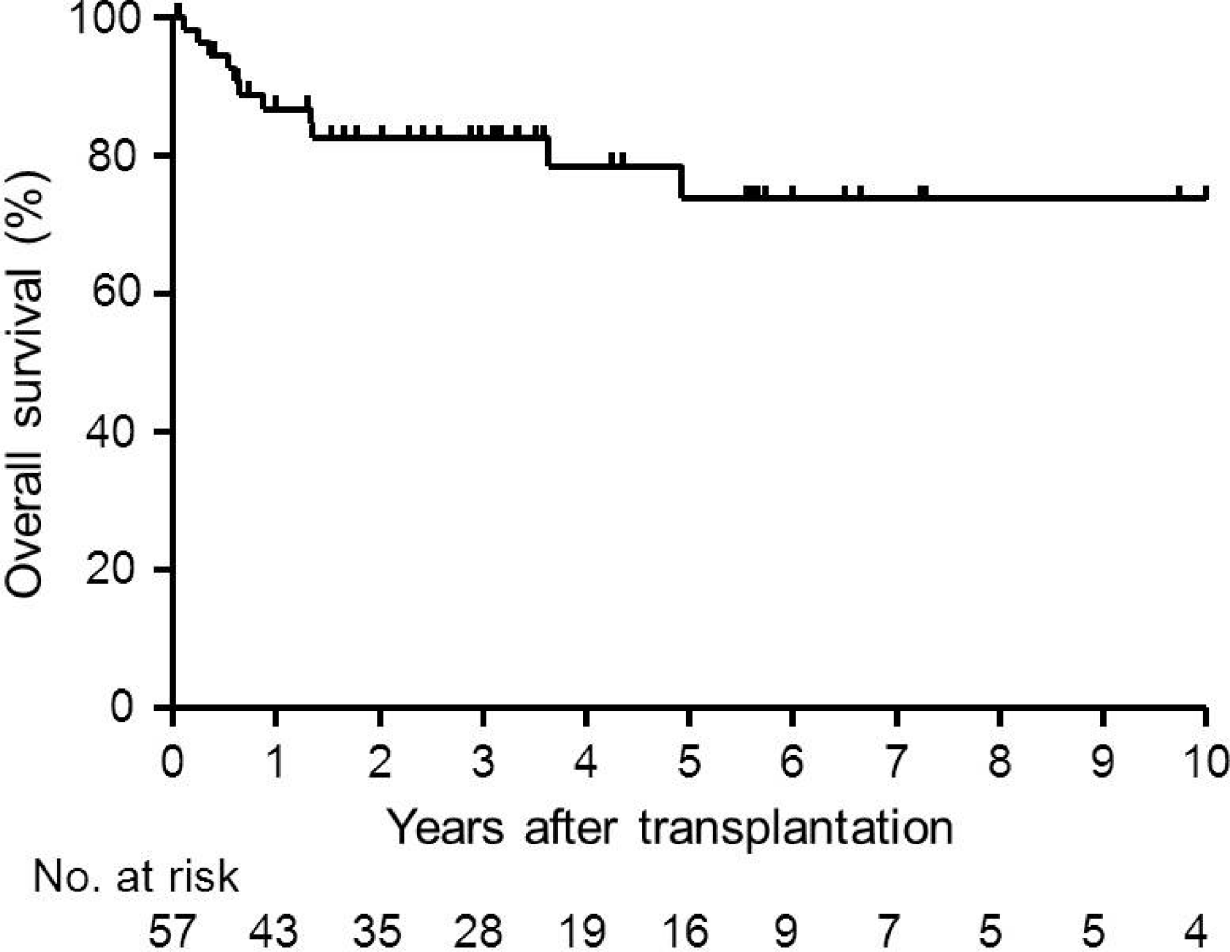
Kaplan-Meier calculated survival rate after lung transplantation for 57 patients with LAM. The median follow-up period was 1,085 days. The probability of survival at 1, 3, 5 and 10 years after lung transplantation was 86.7%, 82.5%, 73.7% and 73.7%, respectively.

The survival rate of 57 transplanted patients was 86.7% at 1 year, 82.5% at 3 years, 73.7% at 5 years, and 73.7% at 10 years (Fig 1). Median follow-up duration from transplantation was 1,085 days (S3 Table). Two patients were re-transplanted for graft failure at 38 months or 73 months after the first transplantation. Eleven (20%) of 57 recipients had died at a median of 237 days after transplantation; six patients died of lung infection, four died of graft failure, and two died of malignant neoplasm.

Survival of all 98 LAM patients after registration was 93.5% at 1 year, 85.9% at 3 years, 81.4% at 5 years, and 65.7% at 10 years (S2 Fig); the patients who had lung transplants were included without censoring. When we compared the LAM patients bearing lung transplants (n = 57) with those awaiting the transplant opportunity (n = 41), the statistical analysis showed no statistically significant difference in the post-registration survival (p = 0.053); 94.7% survived at 1 year, 91.1% at 3 years, 84.9% at 5 years, and 73.0% at 10 years vs. the waiting group’s 91.8%, 75.6%, 75.6%, and 33.6%, respectively, at each corresponding year (S2 Fig). However, we hypothesize that with larger numbers of patients, a statistically significant difference in survival may be detected since p-value is so close to the significant level.

The following LAM-related complications were noted within one year after transplantation: pneumothorax of native lung in seven patients (12%) and newly developed chylothorax or chylous ascites in 12 (21%) and four patients (7%), respectively. As compared with pneumothorax, chylous effusion tended to appear earlier after transplantation, presenting in some patients within one week. The recurrence of LAM in the transplanted lung, which was histologically diagnosed by transbronchial lung biopsy (TBLB), was found in four patients (Table 4). In this study, no procedural data were collected such as intra-operative bleeding, operation time, operation-related complications, etc. during transplantation.

**Table 4:** Summaries of studies on lung transplantation for LAM

	Multi-center studies					Single-center studies	
Study	Boehler A, et al.^6)^	Kpodonu J, et al. ^15)^	Reynaud-Gaubert M, et al. ^16)^	Benden C, et al. ^17)^	Ando K, et al. (This study)	Pechet TT, et al. ^18)^	Machuca TN, et al. ^19)^
Country	Switzerland	U.S.	France	Europe	Japan	U.S.	Brazil
Period	1992 to 1995	1987 to 2002	1988 to 2006	1997 to 2007	2000 to 2014	1989 to 2001	1989 to 2009
No. of patients	34	79	44	61	57	14	10
No. of centers	16	31	9	21	6	1	1
Age at onset (mean)	29	-	33	-	31.9	-	-
Age at diagnosis (mean)	34	-	36	34.4	34.0	35.7	-
Age at transplan-tation (mean)	40	41.1	41	41.3	41.8	41.8	43.8
FEV_1_%pred (mean)	24	-	22.8	27	32.7	20	32.9
DL_co_ %pred (mean)	26	-	27.2^a^	26	24.7	23	38
PaO_2_ (mean), mmHg	56	-	52.8	(59.3)^b^	54.2	53.7	-
6MWD (mean), m	-	-	214	220	248	(251)^c^	-
Mean waiting time (SD)	-	448 days (322)	-	-	1,060 days (649)	1.9 year (1.0)	-
Survival rate	69% (1y) 58% (2y)	85.8% (1y) 76.4% (3y) 64.9% (5y)	79.6% (1y) 74.4% (2 y) 64.7% (5y) 52.4% (10y)	79% (1y) 73% (3y)	86.7% (1y) 82.5% (3y) 73.7% (5y) 73.7% (10y)	100% (1y) 90% (2y) 69% (5y)	90% (1y) 80% (3y)
Recurrence of LAM (No. of patients)	1	-	2	4	4	1	-

^a^ the value is DLco /VA %pred.

^b^7.9 kPa or

^c^826 ft was indicated in each report, respectively.

### Clinical features of LAM patients with a history of inactive status

We then analyzed the clinical features of LAM patients (n = 21) who had a history of inactive status while on the waiting list or were inactive as of March 2014 and compared them with those of LAM patients never self-determined in an inactive status (n = 77). There was no statistically significant difference in age, results of pulmonary function tests, 6MWD, or results of arterial blood gas analysis. However, patients with an inactive history were more frequently treated with sirolimus than those without an inactive history (67% vs. 5%, p<0.001) (S4 Table).

**Fig 2:**
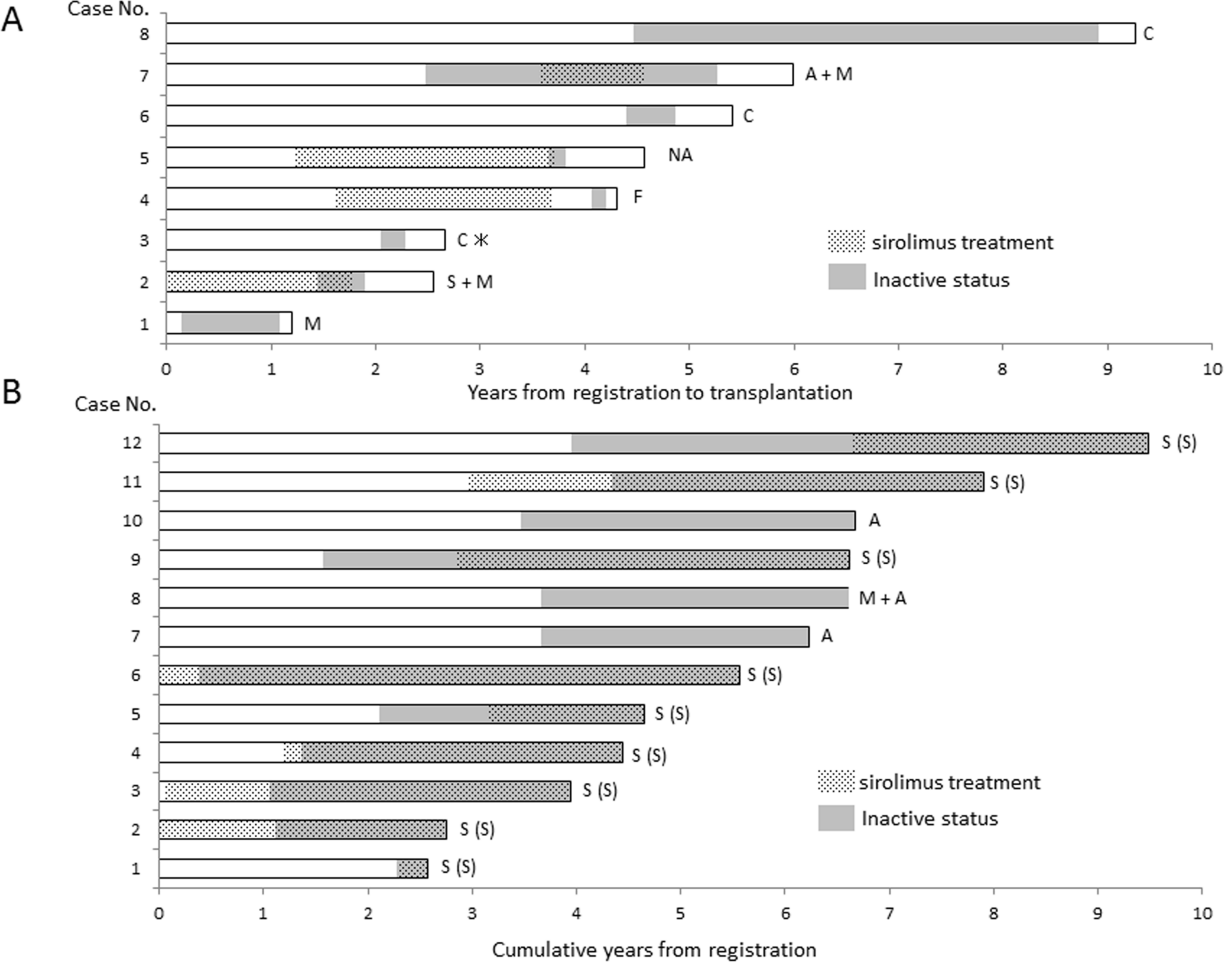
Illustration of the periods of inactive status and sirolimus treatment while on the transplantation waiting list. A. Eight of 21 LAM patients with a history of inactive status had received lung transplants. Five of eight patients had a period during which they had been on the sirolimus treatment (we could not confirm the period of time in case 3*). The reason why a patient had been inactive is indicated at the right end of each illustration by: A, anxiety and fear about transplantation; C, comorbidity (cases 6 and 8, psychiatric disorder and case 3, thyroid tumor); F, family matter; M, participated in the MILES trial; NA, not available; and S, on sirolimus treatment. Although the cases 1 and 7 participated in the MILES trial, they were in the placebo group; the case 7 took sirolimus after the MILES trial. The clinical courses of cases 2, 4, 5, and 7 who took sirolimus, but their clinical courses were not stabilized sufficiently. B. Of twelve patients who were in an inactive status as of March 2014, nine initiated sirolimus treatment before registration (cases 2 and 6) or while on a waiting list (cases 3, 4, 5, 8, 9, 11, and 12). The reason for being inactive is presented with the same manner as in Fig 2A (A and M); S(S) indicates that a patient has been on sirolimus treatment and respiratory condition has improved or been stabilized.

Of twenty-one patients with an inactive history, eight had already received the transplantation, but the remainders were still inactive as of March 2014. One patient died of respiratory failure during waiting term (Table 3). She had been inactive because of anxiety and fear about operation, but returned to be active after 1,598 days of inactive status because of deterioration of her respiratory condition. However, she had no opportunity of receiving transplantation. Of the eight patients given lung transplants, five had a history of sirolimus treatment while on the waiting list (Fig 2A); three (cases 1 and 7, placebo group; case 2, sirolimus group) participated in the MILES trial [10] and five (cases 2, 3, 4, 5, and 7) were on the off-label use of sirolimus; we could not confirm the period of time in case 3. However, their respiratory conditions had not been stabilized, and then they returned to be active. Three patients (cases 3, 6, and 8) became inactive because of their comorbidities (psychiatric disorders [n = 2], thyroid tumor [n = 1]). Meanwhile, nine of the twelve patients who retained the inactive status as of March 2014 began the treatment with sirolimus before or during the wait-list period, and all these patients had continued the treatment thereafter. Their respiratory conditions have improved or been stabilized while on sirolimus and then they have still stayed inactive. The remaining three patients without a history of sirolimus have been inactive because of anxiety and fear about operation; one patient participated in the MILES trial, but she was in the placebo group.

## Discussion

Our study, the first national survey of lung transplantation for LAM patients in Japan, revealed that this group’s survival rate after transplantation was more favorable than that of patients with other indications [7, 8] and also better than recorded in the registry of The International Society for Heart and Lung Transplantation [7]. The better survival statistics of LAM patients after transplantation was previously proven in four multi-center studies and two single-center studies [6, 14–19] (Table 4). Estenne et al. first reported successful lung transplantation for a patient with LAM in 1984 [14]. Thereafter, Boehler et al. described a retrospective multicenter series in 1996 but indicated that disease-related complications were frequent [6]. We found that the age at onset of LAM, diagnosis of LAM, or lung transplantation, DL_CO_ %predicted, PaO_2_, and 6MWD at registration were quite similar among these studies. However, FEV_1_% predicted at registration appears to be better and the waiting period was longer in our population than in those other multi-center studies. The latter issue, the longer waiting time until transplantation, is worthy of special note. This delay was caused primarily by a shortage of donor lungs in Japan. However, an important indicator from this survey is that LAM patients, even those at an advanced stage of disease, can endure until the opportunity for transplantation arrives. Moreover, a favorable survival time is likely once they receive the transplanted lung. This notion is further supported by the fact that the number of LAM patients who died while on the waiting list was only nine (9%).

The occurrences of LAM-related complications in our survey were also similar to those of other studies. In three multi-center studies, the incidences of pneumothorax in native lungs, chylothorax and ascites were 7–14%, 7–14%, and 2%, respectively [6, 16, 17]. We found these complications in 12%, 21% and 7% of patients. The recurrence of LAM in donor lungs is a peculiar post-transplant complication of LAM and also resembled that in other studies (Table 4). The cumulative results of our study confirm that lung transplantation is a satisfactory therapeutic approach for end-stage LAM in Japan, as also found in multiple countries, and this strategy has prolonged life even though LAM is a neoplastic disease.

We estimated that the prevalence of PH at registration for transplantation would be 16 (16%) of 98 LAM patients in our survey judging by echocardiography (SPAP ≥ 40 mmHg) based on the correlation between the results of RHC (MPAP ≥ 25 mmHg) and of echocardiography (SPAP ≥ 40 mmHg). Taveira-DaSilva et al. reported that the estimated SPAP on echocardiography > 35 mmHg was observed in less than 10% of 95 LAM patients [20], but FEV_1_%predicted and PaO_2_ in their subjects were 71.4% and 78.4 Torr, respectively, values that were much milder than those in our population (32.8% and 55.7 Torr, respectively). Our previous quantitative CT analysis demonstrated that cystic lung destruction did not necessarily accompany a concomitant loss of pulmonary vasculature in LAM [21]. Overall, it would be fair to say that PH is prevalent in less than 20% of LAM patients at their registration for lung transplantation.

The MILES trial proved that sirolimus can inhibit the decline of pulmonary function and stabilize the disease in LAM patients whose FEV_1_ is less than 70% of the predicted (mean, 45 ± 10% of the predicted) [10]. This is likely to be true in patients with advanced LAM who are prospective candidates for lung transplantation. Our study revealed that sirolimus treatment significantly alleviated the disease course in LAM patients with a history of inactive status (p <0.001) as compared to those without such a history. Additionally, patients who had received sirolimus therapy (n = 18) might have prolonged survival after registration compared to those who had never received sirolimus (p = 0.073, S2 Fig). These data suggest that sirolimus would have a role in stabilizing disease even in end-stage LAM patients. Furthermore, several case reports in the literature document the beneficial role of sirolimus in post-transplant management of LAM patients; sirolimus ameliorates the post-operative chylothorax [22, 23], extrapulmonary manifestations [24], and recurrence of LAM [25, 26]. In this context, the use of sirolimus is likely to prevail for patients with LAM in need of lung transplantation as a contributor to better prognosis. In addition, sirolimus may have a role in the management of LAM-related complications that occur in the post-transplantation period.

The current uncertainty on the use of sirolimus is how to manage LAM patients who are active candidate for transplants and taking sirolimus. Since bronchial anastomosis dehiscence, a potentially fetal complication of lung transplantation, is reported when a sirolimus-based immunosuppressive therapy was initiated immediately after transplantation [27, 28]. In our cohort, six of 57 transplants (4 with inactive history) was on sirolimus treatment (average, 21 months; range 7 to 37 months) while awaiting transplantation, but were free of sirolimus before transplantation (average, 22 months; range 7 to 43 months). No occurrence of bronchial anastomosis dehiscence was documented in our cohort. At present, all lung transplantation centers in Japan consider to be mandatory for LAM patients to quit taking sirolimus prior to lung transplantation, although evelorimus, another mTOR inhibitor, may be safe to be administered up until lung transplantation [29].

In conclusion, our national survey has revealed that the prognosis after registration as well as that after lung transplantation are favorable in LAM. Lung transplantation is a satisfactory therapeutic option for those with advanced LAM, and the greater use of sirolimus is expected to improve the circumstances of pre-transplantation life for patients with LAM.

## Supporting Information

S1 Fig The correlation between SPAP estimated by echocardiography and MPAP measured by RHC (n = 13). They were positively correlated (r = 0.589, p = 0.034). Six patients whose estimated SPAP was ≥ 40 mmHg had MPAP≥ 25 mmHg by RHC.
(TIF)

S2 Fig Kaplan–Meier calculated survival rate after registration. A. Survival of all 98 LAM patients after registration was 93.5% at 1 year, 85.9% at 3 years, 81.4% at 5 years, and 65.7% at 10 years (solid line); transplantation is not censored. Survival rates of 57 LAM patients who had lung transplantation (coarsely-dotted line) were 94.7%, 91.1%, 84.9%, and 73.0%,whereas those of 41 patients not transplanted (finely-dotted line) were 91.8%, 75.6%, 75.6% and 33.6% at each corresponding year. B. The solid line indicates survival rates after registration of 18 LAM patients: includes those who had sirolimus treatment while waiting for transplantation (n = 9) and also those who had received sirolimus as of March 2014 (n = 9). No patients died while on the waiting list. Of 80 LAM patients who had never received sirolimus, survival after registration was 95.7% at 1 year, 87.5% at 3 years and 78.7% at 5 years (n = 80, dotted). Recipients of sirolimus tended to have better survival rates after registration than those without sirolimus treatment (p = 0.073).
(TIF)

S1 Table Clinical characteristics of 98 LAM patients at the diagnosis of LAM. (DOCX)

S2 Table Correlation between MPAP and other clinical parameters. (DOCX)

S3 Table Outcomes of 57 LAM patients with lung transplantation. (DOCX)

S4 Table Comparison between patients with and without a history of inactive status. (DOCX)
