# Supplementary figures and images for: Lung Transplantation for Lymphangioleiomyomatosis in Japan
Source: PLoS One. 2016 Jan 15;11(1):e0146749. doi: 10.1371/journal.pone.0146749 (PMC4714890; doi:10.1371/journal.pone.0146749)

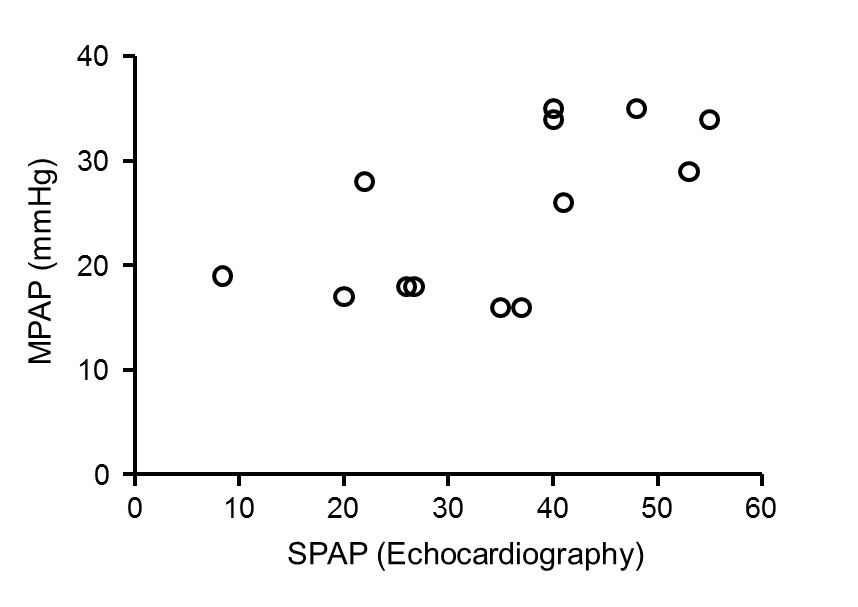

Supplement: S1 Fig — They were positively correlated (r = 0.589, p = 0.034). Six patients whose estimated SPAP was ≥ 40 mmHg had MPAP≥ 25 mmHg by RHC. (TIF) [file pone.0146749.s001.tif]

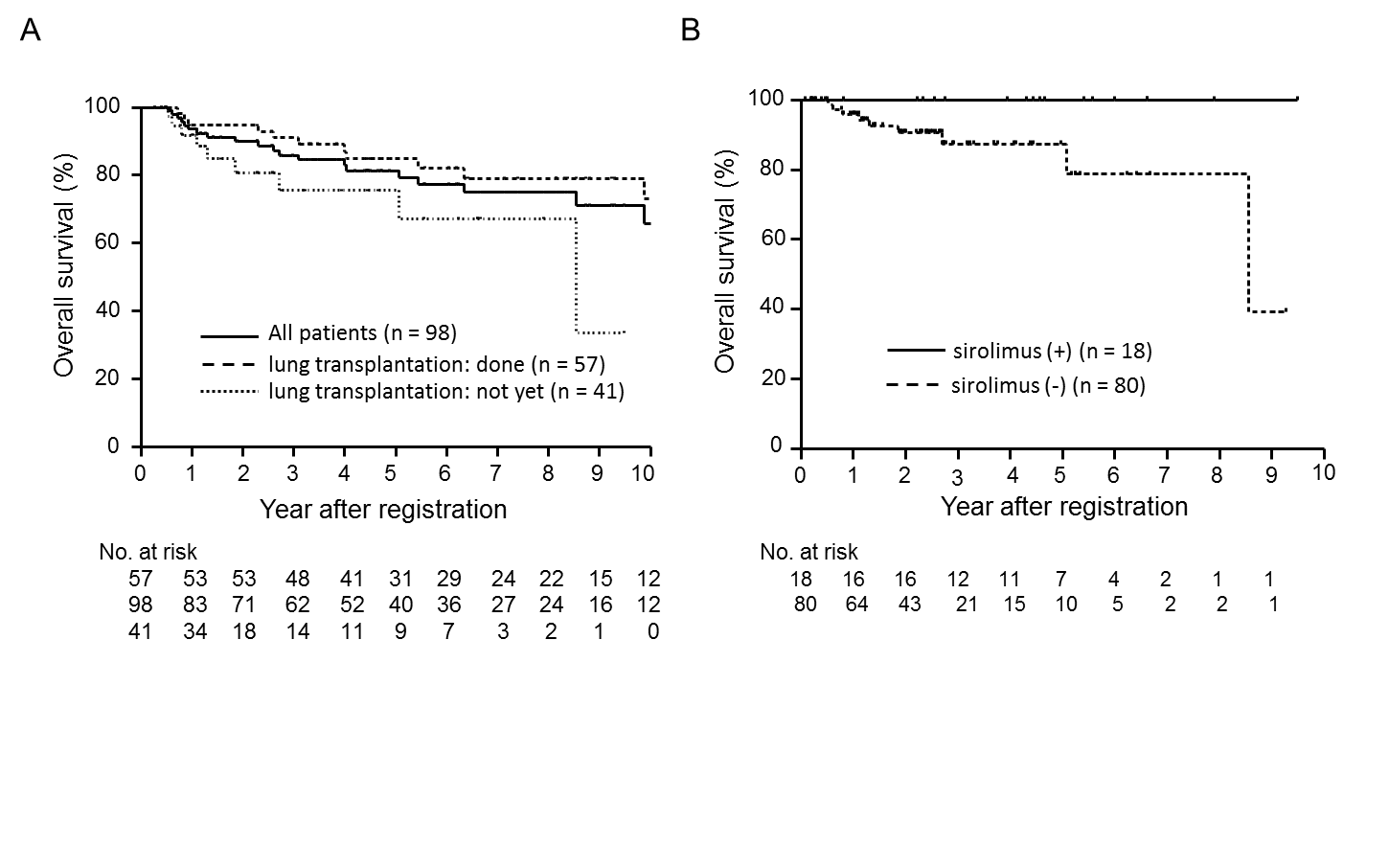

Supplement: S2 Fig — A. Survival of all 98 LAM patients after registration was 93.5% at 1 year, 85.9% at 3 years, 81.4% at 5 years, and 65.7% at 10 years (solid line); transplantation is not censored. Survival rates of 57 LAM patients who had lung transplantation (coarsely-dotted line) were 94.7%, 91.1%, 84.9%, and 73.0%,whereas those of 41 patients not transplanted (finely-dotted line) were 91.8%, 75.6%, 75.6% and 33.6% at each corresponding year. B. The solid line indicates survival rates after registration of 18 LAM patients: includes those who had sirolimus treatment while waiting for transplantation (n = 9) and also those who had received sirolimus as of March 2014 (n = 9). No patients died while on the waiting list. Of 80 LAM patients who had never received sirolimus, survival after registration was 95.7% at 1 year, 87.5% at 3 years and 78.7% at 5 years (n = 80, dotted). Recipients of sirolimus tended to have better survival rates after registration than those without sirolimus treatment (p = 0.073). (TIF) [file pone.0146749.s002.tif]
