# Supplementary material for: Lung Transplantation for Lymphangioleiomyomatosis in Japan
Source: PLoS One. 2016 Jan 15;11(1):e0146749. doi: 10.1371/journal.pone.0146749 (PMC4714890; doi:10.1371/journal.pone.0146749)
Supplement: S1 Table — (DOCX) [file pone.0146749.s003.docx]

**Supplementary Table S1. Clinical characteristics of 98 LAM patients at the diagnosis of LAM**

| Characteristics |  |
| --- | --- |
| Age at onset of symptom –yr (range) | 32.1 ± 7.3 (16 - 52) |
| Age at diagnosis –yr (range) | 34.2 ± 7.5 (18 - 57) |
|  |  |
| Sporadic / TSC-associated LAM -n (%) | 90 (92) / 8 (8) |
| Smoking status |  |
| Ever smoker - n (%) | 27 (28) |
| Smoking index of ever smokers (pack-years) | 6.0 ± 4.5 |
|  |  |
| Presenting features – n　(%) |  |
| Pulmonary manifestations | 92 (93) |
| Pneumothorax | 26 (27) |
| Dyspnea | 50 (51) |
| Cough | 5 (5) |
| Hemoptysis | 3 (3) |
| Chylothorax | 2 (2) |
| Abnormal shadow ^∗^ | 6 (6) |
| Extrapulmonary manifestations | 6 (6) |
|  |  |
| Diagnosis - n (%) |  |
| Lung biopsy | 75 (77) |
| TBLB | 14 (14) |
| VATS | 52 (53) |
| Open lung biopsy | 9 (9) |
| Resection of extrapulmonary lymphangioleiomyoma | 6 (6) |
| Cytology of chylous fluid | 1 (1) |
| Clinical diagnosis ^∗∗^ | 16 (16) |

Plus-minus values are means ± SD.

^∗^ Abnormal shadow was pointed out by chest x-ray which was performed at health screening.

^∗∗^ Diagnosed by the characteristic computed tomography findings and/or elevated serum VEGF-D (> 800 pg/mL).

Abbreviations used are: TBLB, transbronchial lung biopsy; TSC, tuberous sclerosis complex; and VATS, video-assisted thoracoscopic surgery.
