# Supplementary material for: Lung Transplantation for Lymphangioleiomyomatosis in Japan
Source: PLoS One. 2016 Jan 15;11(1):e0146749. doi: 10.1371/journal.pone.0146749 (PMC4714890; doi:10.1371/journal.pone.0146749)
Supplement: S2 Table — (DOCX) [file pone.0146749.s004.docx]

**Supplementary Table S2. Correlation between MPAP and other clinical parameters.**

|  | r | P value |
| --- | --- | --- |
| Age | -0.077 | 0.720 |
| PaO_2_ | -0.348 | 0.223 |
| FVC (%predicted) | 0.225 | 0.291 |
| FEV_1_/FVC | 0.171 | 0.425 |
| FEV_1_ | 0.287 | 0.174 |
| FEV_1_ (%predicted) | 0.283 | 0.181 |
| DL_CO_ (%predicted) | -0.654 | 0.001 |
| 6-min walking distance | 0.361 | 0.091 |
| Cardiac index | -0.145 | 0.498 |

MPAP was determined by right heart catheterization in 25 patients with LAM and then we examined if clinical variables correlated with MPAP.

Abbreviations used are: DL_CO_, carbon monoxide diffusing capacity; FEV_1_, forced expiratory volume in one second; FVC, forced vital capacity; PAP, pulmonary artery pressure; PaO_2_, arterial oxygen tension; and %predicted, a percentage of the predicted values.
