# Supplementary material for: Lung Transplantation for Lymphangioleiomyomatosis in Japan
Source: PLoS One. 2016 Jan 15;11(1):e0146749. doi: 10.1371/journal.pone.0146749 (PMC4714890; doi:10.1371/journal.pone.0146749)
Supplement: S3 Table — (DOCX) [file pone.0146749.s005.docx]

**Supplementary Table S3. Outcomes of 57 LAM patients with lung transplantation**

| Variable |  |
| --- | --- |
| Days from lung transplantation -median (range) | 1,085 (20 - 5,115) |
|  |  |
| Overall survival |  |
| One-year | 86.7 % |
| Three-year | 82.5 % |
| Five-year | 73.7 % |
| Ten-year | 73.7 % |
|  |  |
| Patients who died after transplantation |  |
| Number of patients -n (%) | 11 (20) |
| Days from lung transplantation -median (range) | 237 (37 - 1,795) |
| Cause of death -n |  |
| Infection^∗^ | 6 |
| Primary graft dysfunction | 4 |
| Cancer (cervical cancer, lymphoma) | 2 |

^∗^One patient died of lung infection (lung abscess) and graft failure.
