# Supplementary material for: Lung Transplantation for Lymphangioleiomyomatosis in Japan
Source: PLoS One. 2016 Jan 15;11(1):e0146749. doi: 10.1371/journal.pone.0146749 (PMC4714890; doi:10.1371/journal.pone.0146749)
Supplement: S4 Table — (DOCX) [file pone.0146749.s006.docx]

**Supplementary Table S4. Comparison between patients with and without a history of inactive status.**

| Status | With  an inactive history  (n = 21) | Without  an inactive history  (n = 77) | P value |
| --- | --- | --- | --- |
| Age at onset of symptom –yr | 30.2 ± 6.5 | 32.6 ± 7.5 | 0.180 |
| Age at diagnosis –yr | 32.1 ± 6.0 | 34.7 ± 7.8 | 0.182 |
| Age at registration  for lung transplantation –yr | 37.1 ± 4.5 | 40.1 ± 7.8 | 0.081 |
|  |  |  |  |
| Lung transplantation |  |  |  |
| Recipients - n (%) | 8 (38) | 48 (62) | 0.080 |
| Age at lung transplantation –yr | 43.6 ± 5.6 | 41.5 ± 8.6 | 0.395 |
|  |  |  |  |
| Pulmonary function (range) |  |  |  |
| FVC (%predicted) | 70.8 ± 21.5 | 70.2 ± 25.1 | 0.937 |
| FEV_1_ / FVC | 44.5 ± 21.3 | 39.0 ± 13.1 | 0.565 |
| FEV_1_ (L) | 0.96 ± 0.51 | 0.82 ± 0.42 | 0.257 |
| FEV_1_ (%predicted) | 36.3 ± 18.6 | 31.9 ± 16.6 | 0.326 |
| DLco (%predicted) | 23.5 ± 10.2 | 25.0 ± 11.6 | 0.782 |
|  |  |  |  |
| Six minutes walking test |  |  |  |
| Distance (m) | 285.5 ± 88.9 | 243.1 ± 88.6 | 0.084 |
|  |  |  |  |
| Arterial blood gas (room air)* |  |  |  |
| PaO_2_ (Torr) (range) | 57.7 ± 9.5 | 55.1 ± 8.6 | 0.431 |
| PaCO_2_ (Torr) (range) | 39.2 ± 5.0 | 38.9 ± 5.4 | 0.889 |
|  |  |  |  |
| Sirolimus treatment | 14 (67) | 4 (5) | <0.001 |

*Arterial blood gas was determined in 17 (with an inactive history) and 54 patients (without an inactive history), respectively
